# Editorial: Mutation-Specific Gene Editing for Blood Disorders

**DOI:** 10.3389/fgeed.2021.761771

**Published:** 2021-09-29

**Authors:** Carsten Werner Lederer, Pietro Genovese, Annarita Miccio, Sjaak Philipsen

**Affiliations:** ^1^ Department of Molecular Genetics Thalassaemia, The Cyprus Institute of Neurology and Genetics, Nicosia, Cyprus; ^2^ Boston Children’s Hospital, Harvard Medical School, Boston, MA, United States; ^3^ Université de Paris, Imagine Institute, Laboratory of Chromatin and Gene Regulation During Development, INSERM UMR, Paris, France; ^4^ Department of Cell Biology, Erasmus University Medical Center, Rotterdam, Netherlands

**Keywords:** Gene Editing, CRISPR/Cas, Prime Editing, Base Editing, Perspective, Hematopathology, Nanoblades, Hoogsteen

Hundreds of inherited blood disorders have been characterized and in their majority are still without effective treatments. Although many of them are, in principle, curable by allogeneic hematopoietic stem cell (HSC) transplantation, limited availability of compatible donors and persistent associated risks of treatment-related morbidity and mortality have prompted the search for alternative cures. As a result, gene therapy treatments have been developed, and for some of the more prominent blood disorders these have reached the clinic and even market approval. Presently, the most advanced approaches are based on *ex vivo* therapy by gene addition or genome editing, with the emerging prospect of *in vivo* therapies (Li et al., 2021a; 2021b), which would avoid myeloablation and associated risks of adverse events in the clinic (Frangoul et al., 2020; BioPharma Dive 2021). Permanent gene addition by semi-randomly integrating vectors poses the inherent risk of insertional mutagenesis and of host gene perturbation in HSCs, especially if promoter-proximal integration is favoured or if strong enhancer/promoter regions are included in the transgene cassette (Cattoglio et al. 2007; Servick, 2021). Therefore, the use of editing tools for gene correction, for targeted gene addition or for targeting of disease modifiers is the basis for an ever increasing number of upcoming therapies. Disruption of disease modifiers with gene editing tools as it is currently applied in clinical trials depends on the existence of therapeutically relevant modifiers for a given disease and requires a fundamental understanding of disease pathology, which for many blood disorders is still absent. In this context, mutation-specific editing would be solely based on knowledge of the underlying causative mutations and would represent the most direct curative approach for the majority of inherited defects. Such therapies targeting specific mutations often suffer from low efficiencies in HSCs, and the development costs can be disproportionate for the usually small number of patients that might benefit from the new therapy.

To address these particular shortcomings of mutation-specific therapy and to enhance efficiency of treatments in general and thus to lower dosage requirements and risks to patients, therapy development for blood disorders needs novel strategies as much as it needs incremental improvements to existing protocols. In this respect, this Special Issue on *Mutation-Specific Gene Editing for Blood Disorders* covers an exceptional selection of contributions on mutation-specific gene editing in particular, and more generally on the development of associated concepts, protocols, technologies and corresponding regulatory and socio-economic conditions. The featured reviews and original articles offer expert perspectives on HSC-based therapies and particularly editing for hemoglobinopathies, on bone marrow failure syndromes and blood malignancies, on editing platforms, pathways and delivery methods, on targeted insertion of therapeutic transgenes and on the assessment of off-target activity of gene editing tools:

Several reviews give independent views on the state of the art and the path ahead for gene editing and therapies based on HSCs. In their perspective on the genetic engineering of HSCs, Klaver-Flores et al. briefly cover HSC biology and gene editing platforms, before evaluating key areas of necessary improvement, the *status quo* of HSC engineering for therapy and the requirement for regulatory harmonization. In a different approach, Ferrari et al. comprehensively cover clinical development of homology-directed repair (HDR)-based editing for blood disorders, from editing platforms over HDR optimization, cyto-/genotoxicity and manufacturing protocols up to the post-treatment molecular follow-up. They also touch on the relationship of gene addition with conventional integrating vectors and the emerging gene editing therapies, and on the challenges of creating safe and efficient advanced therapies that are also financially sustainable. With further emphasis on clinical translation, Papanikolaou and Bosio review the current efforts and regulatory frameworks in the commercialization of advanced therapies, and provide an insightful perspective on the ongoing improvements to safety and efficacy, required for successful introduction of gene editing-based therapies.

Paradigmatic for application of editing to HSCs is the prolific and clinically most advanced field of gene editing for hemoglobinopathies. In their review covering both α- and β-hemoglobinopathies, Zittersteijn et al. include mechanisms underlying globin expression, introductory to an up-to-date view on gene addition and on gene editing approaches and platforms for treatment of hemoglobinopathies. Insights into globin regulation are expanded by Mussolino and strouboulis, whose review places particular emphasis on regulatory complexes and therapeutic targets for epigenetic treatment of β-hemoglobinopathies. With focus on primary sequence editing for β-hemoglobinopathies, Barbarani et al. give an overview of editing strategies employed for treatment and point out the significance of reproducing naturally occurring therapeutic modifier mutations, of DNA double-strand break (DSB)-independent editing technologies, and of finding a suitable balance between efficiency and the level of precision required for editing. In this context, original research by Benitez et al. shows how modification of DNA repair factors and in particular their tethering to Cas9 can shift the balance between different DSB repair mechanisms, and thus editing outcomes, for the mutation causing sickle cell disease.

Controlling repair outcomes is critical for the safety and efficacy of localised sequence modifications, but also for the targeted insertion (TI) of therapeutic transgenes, which combines the safety of gene editing with key benefits of gene addition, including the ability to address multiple mutations with one and the same therapeutic tool. In their review, Pavani and Amendola illustrate the extreme versatility of the TI approach, which is based on the insertion of promoterless transgenes behind endogenous promoters or on the insertion of promoter-driven transgenes in safe-harbor loci. TI enables a range of applications, including the deactivation of disease modifiers and the delivery of chimeric antigen receptors (CAR) in the production of CAR cells for adoptive immunotherapy.

The alternative precision editing technologies of base and prime editors are then comprehensively assessed for their initial and latest developments, for their advantages and disadvantages and for their specific applications to blood disorders by Antoniou et al.

Taken together, research into shifting the balance of different DSB repair pathways towards precision edits and for TI, or into the employment of DSB-free precision editors has bearing on many diseases. As Koyunlar and de Pater point out, these include inherited bone marrow failure syndromes caused by GATA2 mutations, of which 90% are found in the open reading frame and thus depend on precision edits, in the absence of a universal modifier as alternative target. Here, the consistent accumulation of additional oncogenic somatic mutations in other genes and the paucity of HSCs as the substrate for gene editing present particular challenges, representative of what holds true also for other blood disorders, such as Fanconi and Diamond Blackfan anemia. Importantly, many blood malignancies are based on and need to be addressed by changes in mesenchymal stromal cells (MSCs) instead of HSCs, as reviewed by Banjanin and Schneider, who in addition to the option of cross-correction of MSCs by modification of HSCs point out the specific challenges of *in vivo* and *ex vivo* editing and validation for MSCs.

For many of the rarest blood disorders, understanding of the underlying biology and availability of disease models are limiting. In this respect the original research article by Simmons et al. demonstrates the utility of CRISPR/Cas9-based editing for the characterization of gene function and the development of *Slc48a1*-mutant mice as putative models for human *SLC48A1*-linked idiopathic iron disorders.

Vectors and delivery technologies are critical to the success of therapies based on gene editing, and the recent achievement of near-saturated *ex vivo* editing in HSCs by electroporation was in large part enabled by advances in synthetic CRISPR/Cas guide RNAs, as reviewed by Allen et al. Pointing out an editing technology alternative to protein and ribonucleoprotein-based editors, Félix et al. address the concept and framework parameters of polypurine reverse Hoogsteen hairpin technology for editing, and its application to a selection of specific mutations causing different blood disorders. As an alternative to the delivery of naked editing molecules, virus-like nanoparticles conceptually enable *ex vivo* and *in vivo* application and make the exploration of editing, also using novel Cas9 flavors, accessible to any laboratory familiar with the production of viral vectors. Gutierrez-Guerrero et al. demonstrate in their primary research article efficient duplex-DSB-based excision and targeted insertion of an adeno-associated-virus–encoded HDR donor into the Wiskott-Aldrich syndrome locus of T, B and CD34^+^ cells by delivery of CRISPR/Cas with virus-like Nanoblades.

For any editing application but even more so for the development of CAR and other technologies potentially drawing on multiple DSBs for editing, off-target activity of editing tools is of particular concern. This point is addressed by Blattner et al., whose review comprehensively surveys biased and unbiased detection technology for off-target activities of different editing platforms, including the issue of translocation detection and remaining challenges, such as robust assays that can be applied in therapeutically relevant cells.

Driven by the rapid and ongoing development of gene editing tools, protocols and applications, editing technology is fast approaching such efficiency, accuracy and ubiquity, that clinically relevant mutation-specific applications for many diseases are within reach. We hope that the original research and authoritative reviews we had the privilege of putting together in this Special Issue will be a further step to making this exciting prospect a reality.
